# ENHANCING CARE FOR OLDER VETERANS WITH COMPLEX PHYSICAL, COGNITIVE, AND PSYCHOLOGICAL COMORBIDITIES

**DOI:** 10.1093/geroni/igad104.1452

**Published:** 2023-12-21

**Authors:** Jeffrey Gregg, Christine Gould, Michele Karel

## Abstract

Older veterans present in healthcare settings with more diagnoses than younger veterans and non-veteran older adults. Older veterans are also more likely to present with co-occurring physical, cognitive, and psychological conditions that are difficult to assess and treat. This is further complicated by the fact that many older veterans reside in rural areas without easy access to specialty care and, even in non-rural settings, there is a dire shortage of providers with geriatric expertise both within and outside of the Veterans Health Administration (VHA) system. The current symposium highlights promising findings from a variety of VHA programs aimed at enhancing the quality and reach of care provided to older veterans with complex needs. First, Dr. Bamonti will present mixed-methods results from her work in the development of Step-Cognitive Behavioral Therapy, a novel virtual intervention for older veterans with COPD. Second, Ms. Carlson will describe program evaluation outcomes from a project aimed at expanding geriatric mental health services via telehealth to older veterans with complex needs residing in rural areas. Third, Dr. Peeples will discuss factors affecting the implementation of the VA Behavioral Recovery Outreach Team model, which is aimed at facilitating successful discharge from inpatient settings and preventing rehospitalization for older veterans. Finally, Dr. Gregg will describe qualitative findings examining a component of the Geriatric Scholars Program for Psychologists, a national post-licensure training program for psychologists treating older rural veterans with complex needs. Dr. Karel, National Director of VHA Geriatric Mental Health, will close with discussion and highlight future directions.

